# The Close of Volume XXVIII

**Published:** 1889-07

**Authors:** 


BUFFALO MEDICAL AND SURGICAL JOURNAL
MONTHLY REVIEW OF MEDICINE AND SURGERY.

EDITORS:
THOS. LOTHROP, M. D., - - - W. W. POTTER, M. D.

All communications, whether of a literary or business character, should be addressed to the editors: 284 FRANKLIN STREET, BUFFALO, N. Y.

### Editorial.

### THE CLOSE OF VOLUME XXVIII.

### COMBINATION WITH THE MEDICAL PRESS OF WESTERN NEW YORK.

THIS July number brings the twenty-eighth volume of the new series of this magazine to its end. There are many reasons for congratulation at this time, and we may be pardoned, we trust, for briefly alluding to some of them.

*First.*—Our subscribers, many of whom have received the JOURNAL since it was originally established in 1845, are already cognizant of the fact that it has been very much increased in size under the present management. We have furnished in this volume more pages than were ever published in a medical periodical in Buffalo before. It is true we made no formal announcement of our intentions in this regard, preferring to give our friends the benefit of whatever increase their support would warrant. Their generosity has been duly appreciated, and the result is before them for critical examination. The comprehensive index that accompanies this number speaks in no uncertain manner upon this point.

*Second.*—We congratulate ourselves on the gradual and steady increase of our subscription list, which has enabled us to accomplish so much ; we hope the future will even yield a more abundant harvest. The interest is mutual ; no medical publication can succeed without a liberal patronage ; no such magazine can deserve success without a generous outlay of time and money.

It is already known to some of our readers that the *Medical Press of Western New York* has become consolidated with the JOURNAL. The editorial announcement in the *Press* for June sufficiently stated the reasons for this action, and we need not repeat them here. This number will be posted to each subscriber to the *Press*, and we will thank any such who may fail to get it promptly if they will notify us to that effect.

To the many friends of the *Press* we will say, that it shall be our aim to deserve, as we expect to receive, their cordial friendship and esteem.

